# Quantitative analysis of choroidal morphology in preeclampsia during pregnancy according to retinal change

**DOI:** 10.1038/s41598-023-40144-2

**Published:** 2023-08-14

**Authors:** In Kee Kim, Jae Eun Shin, Min Jeong Kim, Ho Ra, Jiwon Baek

**Affiliations:** 1 Department of Ophthalmology, College of Medicine, Bucheon St. Mary’s Hospital, The Catholic University of Korea, #327 Sosa-Ro, Wonmi-Gu, Bucheon, Gyeonggi-Do 14647 Republic of Korea; 2 Department of Obstetrics and Gynecology, College of Medicine, Bucheon St. Mary’s Hospital, The Catholic University of Korea, Gyeonggi-Do, Republic of Korea; 3 College of Medicine, The Catholic University of Korea, Seoul, Republic of Korea

**Keywords:** Retinal diseases, Uveal diseases, Reproductive disorders

## Abstract

We sought to investigate changes in choroidal hemodynamics in preeclampsia according to presence of retinal change by quantitatively assessing choroidal vessels using optical coherence tomography (OCT). This retrospective study included 106 eyes (of 53 patients) with preeclampsia, including 70 eyes without retinal change in patients with preeclampsia (Group A), 22 eyes with retinal change in patients with preeclampsia (Group B), and 14 eyes of normal pregnant women (controls). Subfoveal choroidal thickness (SFCT) was measured on OCT B-scan images, and choroidal vessel densities (CVDs) were calculated using binarized OCT B-scan and *en face* images. Their values were then correlated with clinical parameters associated with preeclampsia. SFCT was greater in Group B compared to Group A or controls (354.32 ± 65.13 vs. 288 ± 55.68 or 277.21 ± 50.08, both *P* < 0.001). CVD on B-scan images was greater in Group B compared to Group A or controls (76.4 ± 4.9 vs. 73.7 ± 5.3 or 71.5 ± 5.1; both *P* ≤ 0.046). CVD on *en face* images was also greater in Group B compared to Group A or controls (64.7 ± 0.8 vs. 63.6 ± 1.5 or 63.3 ± 1.3; both *P* ≤ 0.001). SFCT and CVD positively correlated with each other (*P* ≤ 0.009) and were greater in patients with blurred vision and vaginal bleeding (*P* ≤ 0.020 for blurred vision and *P* ≤ 0.024 for vaginal bleeding). SFCT and CVDs were higher in preeclampsia patients with retinal change compared to those without retinal change or controls. Both SFCT and CVD showed association with blurred vision and vaginal bleeding.

## Introduction

Preeclampsia is a pregnancy-specific multisystemic disorder that causes changes in systemic vasculature and hemodynamics, leading to hepatic failure, proteinuria, neurologic symptom, and pulmonary edema^1^. Approximately 30%–40% of patients with preeclampsia have subjective visual disturbance, and various associated ocular findings are observed^2,3^.

Retinal vascular abnormalities can be found frequently in preeclampsia pregnancies^4,5^. Some studies have reported that choroidal ischemia and retinal circulation disorders are present in 30%–100% of preeclampsia pregnancies^6^. Serous retinal detachment (SRD) is a common cause of visual disturbances in preeclampsia. Alterations in choroidal circulation triggered by elevated blood pressure may cause choroidal thickening and subretinal fluid accumulation in preeclampsia and malignant hypertension, causing SRD^7^.

This study aimed to investigate changes in choroidal hemodynamics in preeclampsia patients with or without retinal change by assessing choroidal vascularity using optical coherence tomography (OCT) B-scan and *en face* images. We then analyzed correlations between choroidal and clinical parameters.

## Results

### Patient demographics and clinical characteristics

In total, 70 eyes (of 35 patients) without retinal change (Group A) and 22 eyes (of 11 patients) with retinal change (Group B) were enrolled. Additionally, the control group consisted of 14 eyes of 7 women with normal pregnancies.

All patients were of Asian descent, and the mean age was 34.62 ± 4.73 (range, 18–46) years. There was no difference in the mean age among groups (*P* = 0.762). The mean logMAR BCVA was worse in Group A compared to the other groups (*P* ≤ 0.004). Systolic and diastolic BPs as well as the urine protein level were highest in Group B, followed by Group A and then the control group (*P* < 0.001, *P* < 0.001, and *P* ≤ 0.007). The demographics and clinical characteristics of each group and their comparisons are summarized in Table 1.

**Table 1:** Patient demographics and clinical characteristics.

Parameters	Controls (n = 14)	Preeclampsia without retinal change (n = 70)	Preeclampsia with retinal change (n = 22)	*P* value^a^	P value^b^	*P* value^c^	*P* value^d^
Age (years, mean ± SD)	35.14 ± 3.01	34.46 ± 4.87	34.82 ± 5.31	0.762	0.495	0.816	0.778
BCVA (logMAR, mean ± SD)	0.01 ± 0.02	0.01 ± 0.03	0.15 ± 0.19	< 0.001	0.878	0.004	0.004
BP systolic (mmHg, mean ± SD)	114.43 ± 9.75	148.94 ± 9.43	165.91 ± 13.25	< 0.001	< 0.001	< 0.001	< 0.001
BP diastolic (mmHg, mean ± SD)	73.71 ± 10.24	92.69 ± 9.52	106.73 ± 14.72	< 0.001	< 0.001	< 0.001	< 0.001
Urine protein (mg, mean ± SD)	0.29 ± 0.38	1.81 ± 1.43	2.5 ± 1.46	< 0.001	< 0.001	< 0.001	0.007
Headache (presence, %)	0	37	36	0.023	< 0.001	0.002	0.949
Blurred vision (presence, %)	0	0	82	< 0.001	n/a	n/a	< 0.001
Dizziness (presence, %)	0	11	0	0.108	0.004	< 0.001	0.004
Vaginal bleeding (presence, %)	0	6	9	0.516	0.045	0.162	0.627
Dyspnea (presence, %)	0	14	9	0.289	0.001	0.162	0.496
Edema (presence, %)	0	26	18	0.093	< 0.001	0.042	0.453
Hemoglobin (g/dL, mean ± SD)	12.17 ± 1.49	12.37 ± 1.21	12.05 ± 2.16	0.901	0.645	0.849	0.518
ESR (mm/hr, mean ± SD)	38.14 ± 24.21	39.49 ± 21.02	46.27 ± 24.16	0.171	0.849	0.334	0.245
CRP (mg/L, mean ± SD)	5.77 ± 4.2	7.86 ± 18.28	9.13 ± 17.24	0.434	0.397	0.390	0.768
D-dimer (ug/mL, mean ± SD)	0 ± 0	2.4 ± 2.93	3.62 ± 3.9	0.169	n/a	n/a	0.208
AST (U/L, mean ± SD)	16.71 ± 7.16	21.71 ± 18.57	20 ± 5.07	0.095	0.094	0.150	0.489
ALT (U/L, mean ± SD)	14.43 ± 5.23	20.11 ± 40.27	13.55 ± 6.15	0.553	0.260	0.648	0.192

*SD* standard deviation, *BCVA* best-corrected visual acuity, *logMAR* logarithm of the minimum angle of resolution, *BP* blood pressure, *ESR* erythrocyte sedimentation rate, *CRP* C-reactive protein, *AST* aspartate aminotransferase, *ALT* alanine transaminase.

^a^Comparison among three groups.

^b^Comparison between controls and preeclampsia patients without retinal change.

^c^Comparison between controls and preeclampsia patients with retinal change.

^d^Comparison between preeclampsia patients without and with retinal change.

In Group B, all eyes showed retinal arteriolar narrowing or tortuosity, 8 eyes (33.3%) showed retinal hemorrhages, 14 eyes (58.3%) showed retinal ischemia visualized as cotton wool spots, and 10 eyes (41.7%) showed serous retinal detachments/exudates.

### Choroidal parameters

There were significant differences in CTs and CVDs among groups (all *P* ≤ 0.005). The mean subfoveal, nasal, and temporal CTs were higher in Group B compared to the other groups (all *P* ≤ 0.004). Meanwhile, there was no significant difference in CT values between Group A and the control group (all *P* ≥ 0.438). The mean CVD on OCT B-scan images was higher in Group B compared to the other groups (all *P* ≤ 0.005); moreover, the mean CVD on *en face* OCT images was also higher in Group B (all *P* ≤ 0.001). Conversely, OCT B-scan and *en face* images did not reveal a significant difference in mean CVD between Group A and the control group (all *P* ≥ 0.270). The choroidal parameters of each group and their comparisons are summarized in Table 2.

**Table 2:** Choroidal parameters.

Parameters	Controls (n = 14)	Preeclampsia without retinal change (n = 70)	Preeclampsia with retinal change (n = 22)	*P* value^a^	*P* value^b^	*P* value^c^	*P* value^d^
Choroidal thickness, nasal (um, mean ± SD)	221.57 ± 47.64	232.79 ± 52.17	279.82 ± 50.16	0.001	0.438	0.002	0.001
Choroidal thickness, subfoveal (um, mean ± SD)	277.21 ± 50.08	288 ± 55.68	354.32 ± 65.13	< 0.001	0.479	< 0.001	< 0.001
Choroidal thickness, temporal (um, mean ± SD)	244.21 ± 28.37	248.91 ± 47.81	293.86 ± 61.06	0.005	0.624	0.002	0.004
Choroidal vessel density, B-scan (%, mean ± SD)	71.52 ± 5.09	73.23 ± 5.33	76.73 ± 4.7	0.005	0.270	0.005	0.005
Choroidal vessel density, en face, Haller (%, mean ± SD)	63.26 ± 1.28	63.64 ± 1.44	64.69 ± 0.82	0.001	0.332	0.001	< 0.001

*SD* standard deviation.

^a^Comparison among three groups.

^b^Comparison between controls and preeclampsia patients without retinal change.

^c^Comparison between controls and preeclampsia patients with retinal change.

^d^Comparison between preeclampsia patients without and with retinal change.

When the choroidal parameters were compared according to the presence of retinal change types, eyes with serous retinal detachments/exudate showed significantly thicker subfoveal CT compared to eyes without (*P* = 0.002). Presence of hemorrhage nor ischemia did not affect either CT or CVD (all *P* ≥ 0.070; Table 3).

**Table 3:** Choroidal parameters regarding presence of retinal changes.

Parameters	CT, subfoveal (um, mean ± SD)	CVD, B-scan (%, mean ± SD)	CVD, en face (%, mean ± SD)
Hemorrhage			
( −)	342.44 ± 62.17	77.27 ± 4.59	64.73 ± 0.94
( +)	363.63 ± 71.65	76.56 ± 5.21	65.15 ± 1.12
*P* value^a^	0.528	0.697	0.742
Ischemia			
( −)	344.50 ± 50.18	78.77 ± 4.53	64.32 ± 0.89
( +)	352.00 ± 72.29	76.16 ± 4.68	65.15 ± 0.96
*P* value^a^	0.742	0.192	0.070
SRD/Exudate			
( −)	315.14 ± 47.29	77.23 ± 4.23	64.95 ± 1.27
( +)	397.60 ± 55.35	76.75 ± 5.53	64.76 ± 0.47
*P* value^a^	0.002*	0.977	0.585

*CT* choroidal thickness, *CVD* choroidal vessel density, *SD* standard deviation, *SRD* serous retinal detachment.

^a^Comparison between ( +) and ( −).

### Correlations among choroidal parameters

**Figure 1:**
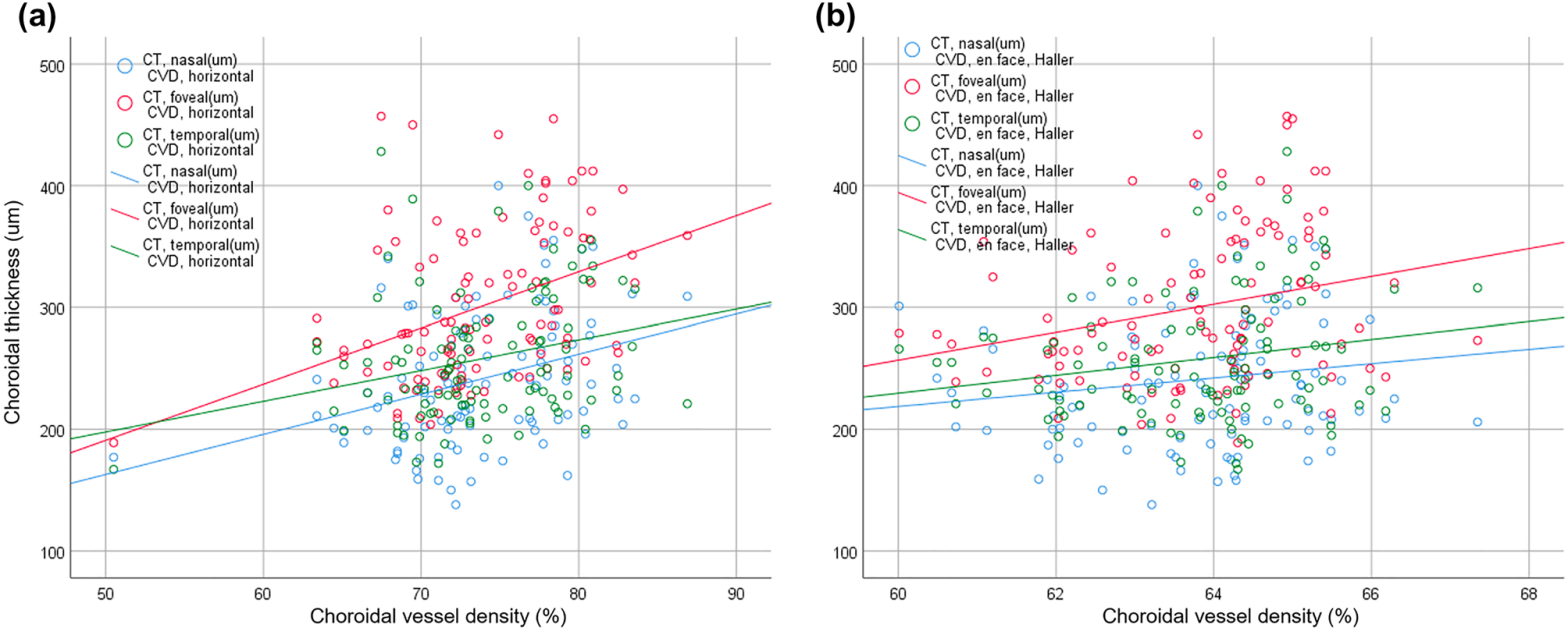
Correlations between choroidal thickness (CT) and choroidal vessel density (CVD). (**a**) A plot of foveal, nasal, and temporal CTs and CVDs on optical coherence tomography B-scan images shows positive associations. (**b**) A plot of foveal and temporal CTs and CVDs on *en face* optical coherence tomography images shows positive correlations.

Overall, foveal, nasal, and temporal CTs showed strong positive correlations with each other (all *P* < 0.001). The CVDs on OCT B-scan images showed positive correlations with the foveal, nasal, and temporal CTs and the CVD on *en face* images (all *P* ≤ 0.007). Meanwhile, the CVD on *en face* OCT images showed positive correlations with foveal and temporal CTs and the CVD on B-scan images (all *P* ≤ 0.044). Correlations among choroidal parameters are summarized in Table 4 and Fig. 1.

**Table 4:** Correlations among choroidal parameters.

Parameters	CT, nasal	CT, foveal	CT, temporal	CVD, B-scan	CVD, en face
CT, nasal					
r	1.000	0.710^a^	0.675^a^	0.325^a^	0.149
*P* value		< 0.001	< 0.001	0.001	0.128
CT, foveal					
r	0.710^a^	1.000	0.809**	0.394^a^	0.251^a^
*P* value	< 0.001		< 0.001	< 0.001	0.009
CT, temporal					
r	0.675^a^	0.809^a^	1.000	0.261^a^	0.196^a^
*P* value	< 0.001	< 0.001		0.007	0.044
CVD, B-scan					
r	0.325^a^	0.394^a^	0.261^a^	1.000	0.515^a^
*P* value	0.001	< 0.001	0.007		< 0.001
CVD, en face					
r	0.149	0.251**	0.196*	0.515^a^	1.000
*P* value	0.128	0.009	0.044	< 0.001	

*Statistically significant value.

*CT* choroidal thickness, *CVD* choroidal vessel density.

^a^Correlation is significant at the 0.05 level (two-tailed).

### Correlations among choroidal parameters and clinical parameters

**Figure 2:**
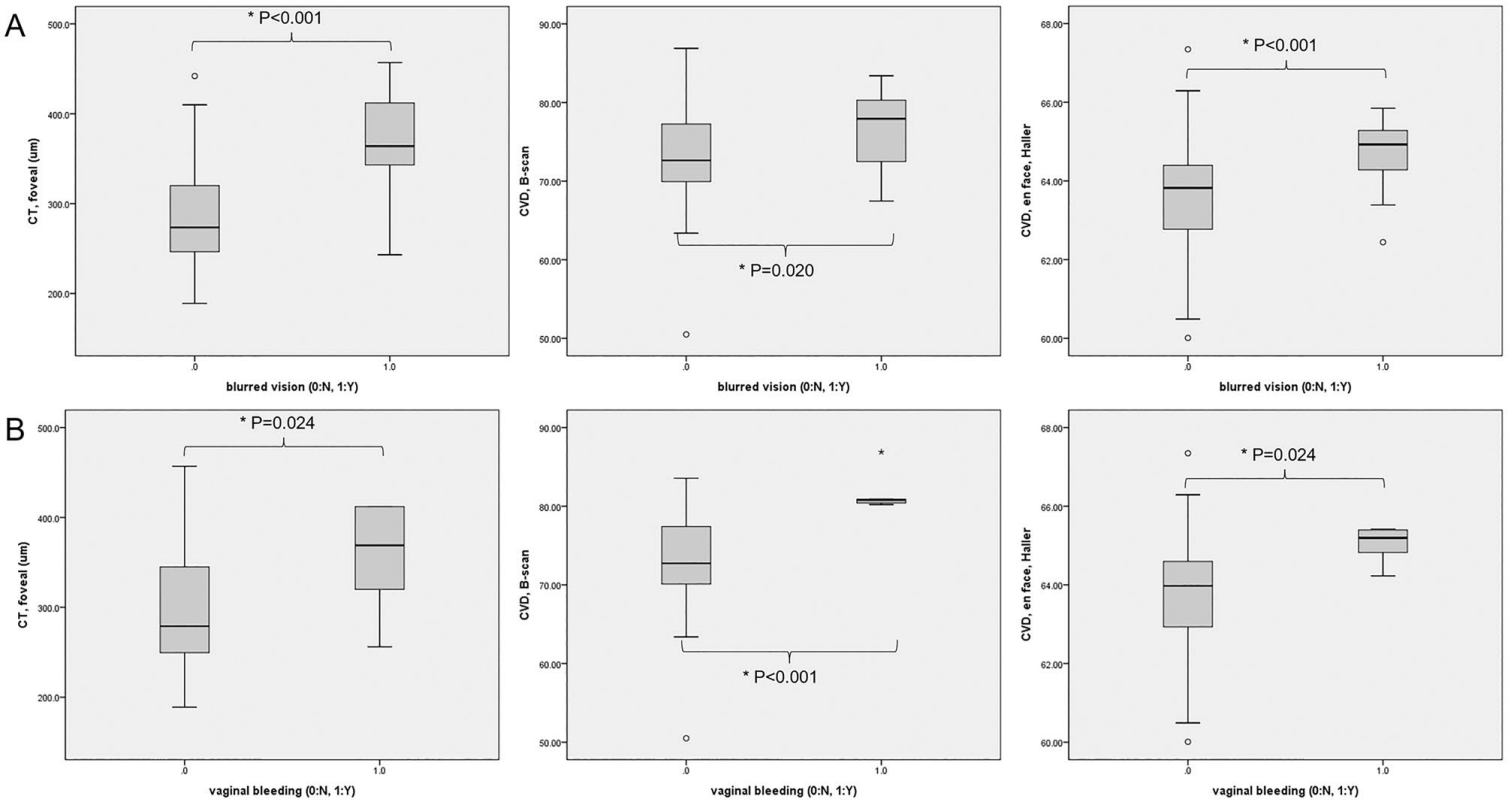
Subfoveal choroidal thickness (CT) and choroidal vessel density (CVD) from optical coherence tomography B-scan and *en face* images in relation to blurred vision and vaginal bleeding. (**A**) CT and CVD values were higher in subjects with blurred vision. (**B**) CT and CVD values were higher in subjects with vaginal bleeding.

The logMAR BCVA positively correlated with foveal and temporal CTs and the CVD on *en face* OCT images (all *P* ≤ 0.037). Systolic BP was positively correlated with foveal and temporal CTs and CVDs on OCT B-scan and *en face* images (all *P* ≤ 0.024). Blurred vision was associated with foveal, nasal, and temporal CTs and CVDs on OCT B-scan and *en face* images (all *P* ≤ 0.020). Vaginal bleeding was associated with foveal and nasal CTs and CVDs on OCT B-scan and *en face* images (all *P* ≤ 0.030). Choroidal parameters correlating with blurred vision and vaginal bleeding are shown in Fig. 2. Correlations among choroidal and clinical parameters are summarized in Table 5.

**Table 5:** Correlations among choroidal parameters and clinical parameters.

Parameters	Age	Visual acuity	BP systolic	BP diastolic	Urine protein	Headache	Blurred vision	Dizziness	Vaginal bleeding	Dyspnea	Edema	Hb	ESR	CRP	D-dimer	AST	ALT
CT, nasal																	
r	− 0.152	0.114	0.298*	0.222*	0.034	0.076	0.330*	− 0.123	0.211*	0.013	− 0.090	0.054	− 0.072	− 0.010	0.145	− 0.072	− 0.061
*P*	0.120	0.243	0.002	0.022	0.733	0.440	0.001	0.211	0.030	0.893	0.356	0.582	0.465	0.920	0.178	0.464	0.536
CT, foveal																	
r	− 0.122	0.292*	0.276*	0.146	0.145	0.150	0.497*	− 0.096	0.218*	− 0.028	0.069	− 0.079	− 0.092	0.028	0.139	− 0.056	− 0.063
*P*	0.213	0.002	0.004	0.134	0.137	0.126	< 0.001	0.328	0.024	0.779	0.484	0.418	0.351	0.773	0.197	0.567	0.518
CT, temporal																	
r	− 0.114	0.292**	0.219*	0.102	0.094	0.046	0.397*	− 0.001	0.108	0.001	0.088	0.047	− 0.089	0.090	0.209	0.037	− 0.013
*P*	0.244	0.002	0.024	0.300	0.337	0.636	< 0.001	0.994	0.272	0.993	0.368	0.634	0.367	0.358	0.050	0.709	0.891
CVD, B-scan																	
r	− 0.108	− 0.039	0.281**	0.188	− 0.062	− 0.049	0.225*	− 0.092	0.363*	− 0.046	− 0.031	0.149	− 0.073	− 0.128	0.021	− 0.011	− 0.033
*P*	0.270	0.688	0.004	0.054	0.527	0.617	0.020	0.349	< 0.001	0.642	0.756	0.127	0.459	0.190	0.844	0.913	0.735
CVD, en face																	
r	− 0.288*	0.203*	0.286*	0.327*	0.221*	− 0.113	0.293*	− 0.163	0.220*	0.206*	0.139	0.213*	− 0.122	− 0.040	0.105	0.023	− 0.064
*P*	0.003	0.037	0.003	0.001	0.023	0.250	0.002	0.096	0.024	0.034	0.155	0.028	0.212	0.686	0.330	0.811	0.515

*BP* blood pressure, *Hb* hemoglobin, *ESR* erythrocyte sedimentation rate, *CRP* C-reactive protein, *AST* aspartate aminotransferase, *ALT* alanine transaminase, *CT* choroidal thickness, *CVD* choroidal vessel density, *r* correlation coefficient, *P*, *P* value.

*Correlation is significant at the 0.05 level (two-tailed).

## Discussion

Changes in the choroid with preeclamptic pregnancies have been studied previously^8–11^. However, most prior studies compared CT values on OCT images, and their results seem controversial. In this study, we analyzed CT values in preeclampsia patients according to the presence of retinal changes and compared patients with one another and with women with normal pregnancies to see whether retinal change is associated with choroidal change. Additionally, the CVD, along with the CT, was measured vertically and horizontally on OCT images to obtain more detailed information about choroidal hemodynamic change in these patients.

Diastolic and systolic BPs as well as the urine protein level were higher in preeclampsia patients with retinal changes compared to those without retinal changes. The BP and urine protein level indicate the severity of preeclampsia; in other words, more severe preeclampsia can be associated with changes in these values. Our results are consistent with those of Kaliaperuma et al., who reported that diastolic BP positively correlated with retinopathy in severe preeclampsia^12^. In addition, Garg et al. documented subclinical retinal and choroidal thickening in the setting of severe preeclampsia^8^.

Foveal, nasal, and temporal CTs were higher in preeclampsia patients with retinal change compared to the other groups. And there was no difference in CTs between preeclampsia patients without retinal change and women in the control group. This suggests that the presence of retinal changes may have affected SFCT results among preeclampsia patients. The discrepancies between previous studies may be explained by differences in disease severity or the presence of retinal changes among participants. It was not possible to analyze the severity of preeclampsia or the presence of retinal changes in previous studies since most did not provide these data. Only Garg et al. reported that differences in CT values between patients with and without visual changes trended toward significance, which was similar to our result^8^.

CVDs on OCT B-scan and *en face* images were also higher in preeclampsia patients with retinal change compared to the other groups, and they did also differ between preeclampsia patients without retinal change and the control group. CVD values also showed a positive correlation with CT values. From this, it can be inferred that increased vascular area in the choroid might be a major factor for choroidal thickening during pregnancy. Previously, Azuma et al. reported that a larger choroidal luminal area was associated with a higher systolic BP in pregnancy^13^. Choroidal overperfusion and increased vascular resistance in a pregnancy-induced hypertension patients with SRD had been demonstrated using laser speckle flowgraphy^14^. Also, SRD was associated with thicker CT in the current study. Regarding a significant positive correlation among ocular blood pressure, choroidal blood flow, and CT, an increase in choroidal vessel luminal area might be caused by the increased volume of circulating blood during pregnancy^15^. In addition, *en face* OCT images revealed a broader macular area compared to OCT B-scan images, suggesting that an increased diameter of Haller vessels is responsible for the choroidal thickening.

Among the clinical parameters associated with preeclampsia, BCVA, systolic BP, and blurred vision and vaginal bleeding correlated with both CTs and CVDs. Again, the correlation between visual disturbances and choroidal parameters is consistent with the previous results of Garg et al.^8^ A high systolic BP suggests a more severe state of preeclampsia; therefore, the severity of the disease seems to be associated with choroidal parameters. Meanwhile, there is a study showing an inverse correlation between vaginal bleeding and preeclampsia earlier in pregnancy and a positive correlation with post-partum bleeding, but there are limited data on the association between vaginal bleeding and preeclampsia severity during late pregnancy^16^, necessitating further investigation.

There are several limitations to this study. First, our sample size was relatively small and only consisted of Asian patients. Also, it has been suggested that CT changes can differ according to the pregnancy trimester^17^. Since this study included pregnant women in their third trimester only, we could not analyze differences in choroidal parameters according to gestation week. Nonetheless, this study evaluated choroidal changes in preeclampsia according to retinal change, which we do not believe any previous study has clearly demonstrated. In addition, not only CT values, but also CVD values on both OCT B-scan and *en face* images were analyzed, proving correlations between CT and CVD values and showing broader areas of choroidal change.

In conclusion, CTs and CVDs were higher in preeclampsia patients with retinal change compared to those without retinal changes or controls. CTs and CVDs correlated with each other and showed an association with blurred vision and vaginal bleeding. We believe that the results of this study have value in explaining discrepancies between previous studies.

## Methods

### Study population

This study was conducted in the Ophthalmology Department at Buchen St. Mary Hospital of The Catholic University of Korea in Gyeonggi-do, Republic of Korea (HC22RASI0091). In this retrospective chart review study, consecutive patients seen between June 2017 and July 2021 who were diagnosed with preeclampsia, hospitalized during their pregnancy, and referred to the ophthalmology department during gestation weeks 32–36 were enrolled. Women with normal pregnancies were age- and gestational age–matched and enrolled as a control group.

Diagnosis of preeclampsia followed the criteria agreed upon by the National High Blood Pressure Education Program Working Group of the U.S. National Institutes of Health, which were blood pressure (BP) ≥ 140/90 mmHg after 20 weeks of gestation on ≥ 2 occasions 6 h apart except for gestational trophoblastic disease or multiple pregnancies, with proteinuria > 0.3 g per 24 h in previously normotensive patients^18^. Preeclampsia patients were divided into two groups according to presence of retinal changes. Retinal changes included focal or generalized arteriolar narrowing, scattered intraretinal hemorrhages and/or ischemia, arterial and venous occlusion, serous retinal detachments, and exudations observed during fundus OCT examination.

Medical records, including systolic and diastolic BPs and symptoms associated with preeclampsia (e.g., headache, blurred vision, dizziness, vaginal bleeding, dyspnea, edema) were reviewed. Laboratory test values for urine protein, serum hemoglobin, erythrocyte sedimentation rate (ESR), C-reactive protein (CRP), D-dimer, aspartate aminotransferase (AST), and alanine aminotransferase (ALT) were also recorded. Finally, collected ophthalmologic examination data included best-corrected visual acuity (BCVA) and OCT images (Cirrus 4000 or Cirrus 6000; Carl Zeiss Meditec, Jena, Germany).

### Image acquisition and analysis

**Figure 3:**
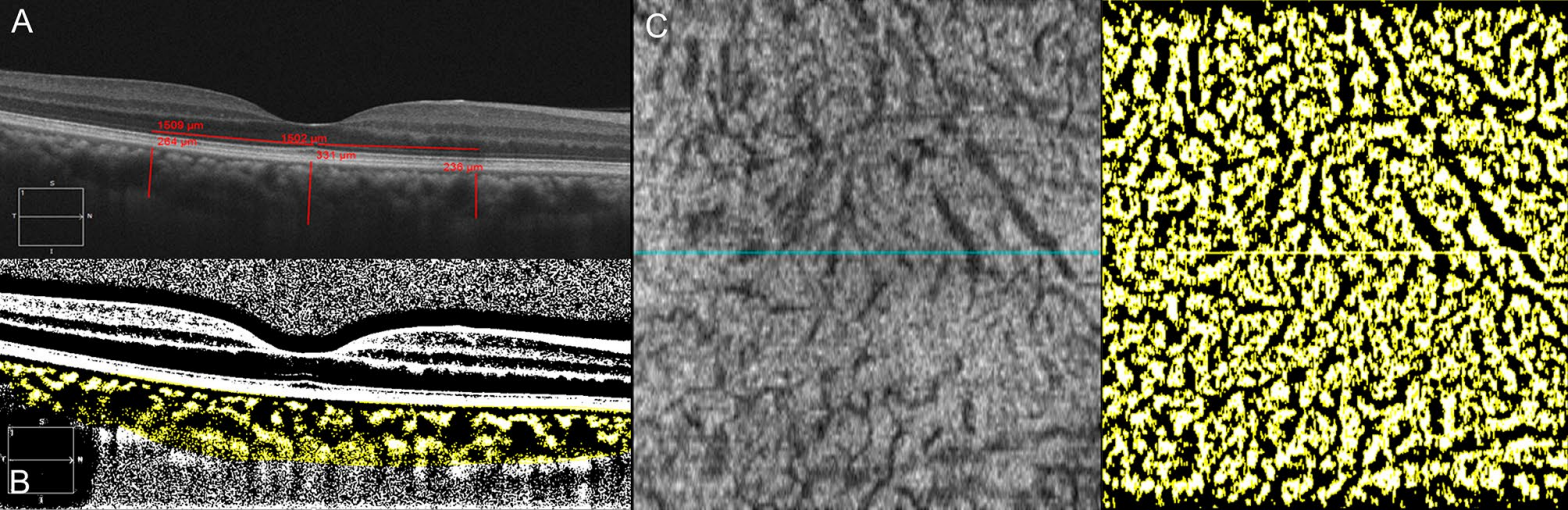
Analysis of optical coherence tomography (OCT) images. (**A**) Choroidal thickness (CT) was measured at three points, i.e., the center and 1500 µm nasally and temporally from the center on HD line scan OCT B-scan images that crossed the fovea. (**B**) Choroidal vascularity (CV) was calculated by dividing the number of pixels in the vascular area (dark pixels) within the choroid by that of the total choroidal area on the OCT B-scan image after binarization. (**C**) A Haller vessel slab was obtained at 50% of the total CT. CV was calculated by dividing the number of pixels in the vascular area (dark pixels) by that of the total area after binarization.

Choroidal thickness (CT) was measured on HD line scan OCT B-scan imager that crossed the fovea. CT was measured at three points, i.e., the center and nasally and temporally 1500 µm from the center. CT was defined using the vertical distance between Bruch’s membrane and the chorio-scleral junction. Figure. 3A Choroidal vessel densities (CVDs) were measured on OCT B-scan images (Fig. 3B) and *en face* image slabs of Haller’s layer (Fig. 3C). A Haller vessel slab was obtained by moving the reference line to a point that was 50% of the total CT. To measure the CVD, binarization of the OCT image was completed using a modified Niblack method with Fiji (available at fiji.sc, free of charge)^19^. The CVD was calculated by dividing the number of pixels in the vascular area (dark pixels) by that of the total choroidal area on the B-scan image and that of the total area on the *en face* image, respectively (Fig. 1C). CTs and CVDs were compared between groups and correlated with other clinical parameters.

### Statistical analysis

Statistical analysis was performed using SPSS for Windows (version 23.0.1; IBM Corporation, Armonk, NY, USA). Mann–Whitney *U* and Kruskal–Wallis tests were used to compare continuous variables among and between groups. Fisher’s least significant difference was used as the post-hoc test after analysis of variance. Categorical variables between groups were compared using the chi-square test, and standardized adjustment was completed as the post-hoc test after the chi-square test. Correlations between variables were accessed using Spearman’s correlation coefficient. *P* < 0.05 was considered statistically significant.

### Ethics approval and consent to participate

This study was conducted in accordance with the tenets of the Declaration of Helsinki. The study was approved by the Institutional Review Board of Bucheon St. Mary’s Hospital, which waived the need for written informed consent because of the study’s retrospective design (HC22RASI0091).

## Data Availability

The datasets generated and/or analyzed during the current study are available from the corresponding author upon reasonable request.
